# Negative Appendicectomy Rate: Incidence and Predictors

**DOI:** 10.7759/cureus.21489

**Published:** 2022-01-22

**Authors:** Khaled Noureldin, Ali Asgar Hatim Ali, Mohamed Issa, Heer Shah, Bolu Ayantunde, Abraham Ayantunde

**Affiliations:** 1 General Surgery, Cairo University Hospital, Cairo, EGY; 2 Colorectal Surgery, Southend University Hospital, National Health Service (NHS) Trust, Essex, GBR; 3 General Surgery, Southend University Hospital, National Health Service (NHS) Trust, Essex, GBR; 4 Surgery, Wirral University Teaching Hospital, Wirral, GBR; 5 Anatomy, University of Dunde, Dunde, GBR

**Keywords:** incidence of negative appendicitis, pain right iliac fossa, emergency appendicectomy, outcome of appendicectomy, predictors of negative appendicectomy, negative appendicectomy

## Abstract

Introduction

Acute appendicitis is a common emergency surgical presentation. The gold standard treatment is surgery. Like any surgical procedure, appendicectomy is associated with complications. Negative appendicectomy (NA) can occur, and its incidence is 15%-39%. This study aimed to evaluate the rate and predictors of NA in a cohort.

Patients and methods

A retrospective study over a year through which data of patients who underwent emergency appendicectomies were collected and analyzed. The absence of inflammatory process and/or other significant pathology in the appendix was considered negative for appendicitis. An utter definition of NA was the absence of inflammatory cells in the appendix. The NA rate (NAR) was calculated using the standard criteria (NAR-SDC) and the strict criteria (NAR-STC). The routine laboratory parameters for diagnosing acute appendicitis on admission were collected. Increased inflammatory markers in the form of leucocytosis of total WBC > 11,000 per mm, elevated CPR > 5 mg/L, and isolated elevated total serum bilirubin > 20 µmol/L, were suggestive of acute appendicitis.

Results

Three hundred and seventy-two patients were included, 179 males and 193 females with a median age were 27 (5-94) years. The median duration of symptoms and waiting time to surgery were two days and one day, respectively. The mean admission WBC, C-reactive protein (CRP) and serum bilirubin levels were 12,600 (3,000-38,000)/mm^3^, 66.9 (1-323) mg/L and 12.7 (4-38) µmol/L respectively. Laparoscopic appendicectomy was performed in 93.5% of patients with a conversion rate of 4.6%. NAR-SDC was 10.2% and NAR-STC was 25.8%. NAR was significantly higher in females than males (39.4% versus 11.1%; p-value 0.0001). Patients with NA were younger (p-value 0.0001), had lower mean total WBC (p-value 0.014), CRP (p-value 0.0001) and total serum bilirubin (p-value 0.0001) levels on admission.

Conclusion

NA is still a major problem in the management of patients with acute right lower abdominal pain. Our NAR compared favourably with reported rates. Female gender, duration of symptoms more than three days, and lower total WBC were independent predictors of NA.

## Introduction

Acute appendicitis is one of the common presentations of acute surgical abdomen worldwide, with a lifetime risk of 7%-8% in the US [1]. The gold standard treatment for acute appendicitis is appendicectomy; however, it is associated with complications and negative appendicectomy [2]. There are reports of antibiotic treatment options in uncomplicated diseases and other carefully selected groups of patients [3]. However, the sole use of antibiotic therapy for uncomplicated acute appendicitis is well inferior to surgery in a randomized controlled trial [4].

Appendicectomy has been associated with negative appendectomy rates (NARs) of 15%-39% in large series. The NAR is well determined by the definition of the term applied in the published study, but unfortunately, there is currently no widely accepted standard definition of a negative appendicectomy [5,6]. Traditionally, negative appendicectomy can be either a macroscopically and/or a histologically normal appendix with no evidence of acute inflammatory process. In its stricter definition, a negative appendicectomy refers to an appendicular specimen in which there are no findings of pathological inflammation as evident by infiltration of the mucosal and/or wall by inflammatory cells, particularly polymorphonuclear leucocytes, lymphocytes, or plasma cells [2]. 

There are reports of declining NARs with 6%-8% reported figures. However, it must be noted that the application of more liberal definitions of negative appendicectomy used in many published reports would automatically lead to lower NARs. It is also evident that many of the published reports from meta-analyses and those based on the national database studies tend to have a vague definition for negative appendicectomy. Some of them based their published data on the discharge letter diagnosis and/or only an intraoperative appearance of the appendix rather than the histological reports [7,8]. 

There is considerable contention and varying opinions on managing a normal appendix found at diagnostic laparoscopy performed for right lower abdominal pain [5]. However, a generally accepted practice is that a macroscopically normal appendix found at laparoscopy is left alone, especially if there is an obvious other reason to account for the patient's symptoms [5,9]. There is arguably reported evidence of increased morbidity, prolonged hospital and their adverse economic implication in patients who have had a normal appendix removed [10]. The other authors who favour removing a normal appendix at laparoscopy argued that such might harbour microscopic appendicitis and/or other pathology responsible for the patient's symptoms [5]. 

We in our centre generally adopt the protocol of removing a macroscopically looking normal appendix found at diagnostic laparoscopy and open operation specifically performed for acute right lower abdominal pain provided there are no other obvious abnormality and/or pathology found in the abdomen and pelvis to account for the patient's symptoms.

The current study aimed to determine the NAR, the predictors of negative appendicectomy, and the outcomes of patients with negative appendicectomy compared with those with histologically confirmed appendicitis.

## Materials and methods

A retrospective study analyzed all emergency appendicectomy procedures performed and the related pathological database over a year from January to December 2019. The research team had to investigate the saved data on the hospital's records to obtain the relevant information for our current study. We collected patients' demographics and clinicopathological data, including the duration of symptoms, comorbidities, American Society of Anaesthesiologists (ASA) class, Charlson comorbidity index (CCI), investigations, operative notes, histopathology reports, hospital stay, and postoperative days complications, discharge summaries, any readmissions and, the follow-up clinical letters.

This study excluded all incidental appendicectomies performed as part of other major emergency or elective procedures not primarily for acute appendicitis and interval appendicectomies. The standard definition of negative appendicectomy is the absence of inflammatory process and/or no other significant pathologic change identified in the appendicular surgical specimen (Standard criteria [SDC]). In its strict definition, negative appendicectomy is the absence of inflammatory polymorphonuclear cells in the appendix muscular wall (Strict criteria [STC]). Based on these definitions, we evaluate and compare the NAR using the standard criteria (NAR-SDC) and the strict criteria (NAR-STC) as outlined above.

The routine laboratory parameters for diagnosing acute appendicitis on admission were collected. Leucocytosis was defined as a total white blood cell (WBC) count of more than 11,000 per mm^3^, elevated C-reactive protein (CRP) of more than 5 mg/L, and an isolated elevated total serum bilirubin level of greater than 20 µmol/L. The findings of the preoperative abdominopelvic ultrasound scan (USS) and the computerized tomography (CT) scan where done were also noted.

Statistical analysis

The collected data were analyzed using IBM SPSS Statistical package version 26.0 (SPSS Inc; Chicago, IL, USA). Continuous and normally distributed variables were expressed in descriptive statistics as mean ± standard deviation (SD). Categorical variables were presented as figures and/or percentages (%). Various variables were compared between the positive and negative appendicectomy groups using the independent Student t-test for normally distributed continuous variables. Pearson Chi-square test was performed to analyze categorical variables. Identified statistically significant variables on univariate analysis for negative appendicectomy were subjected to a logistic regression model to identify the independent predictors of negative appendicectomies. Data analysis was expressed with a 95% confidence interval (CI) and odds ratio (OR). Any variables achieving a p-value less than 0.05 were considered statistically significant.

## Results

**Figure 1:**
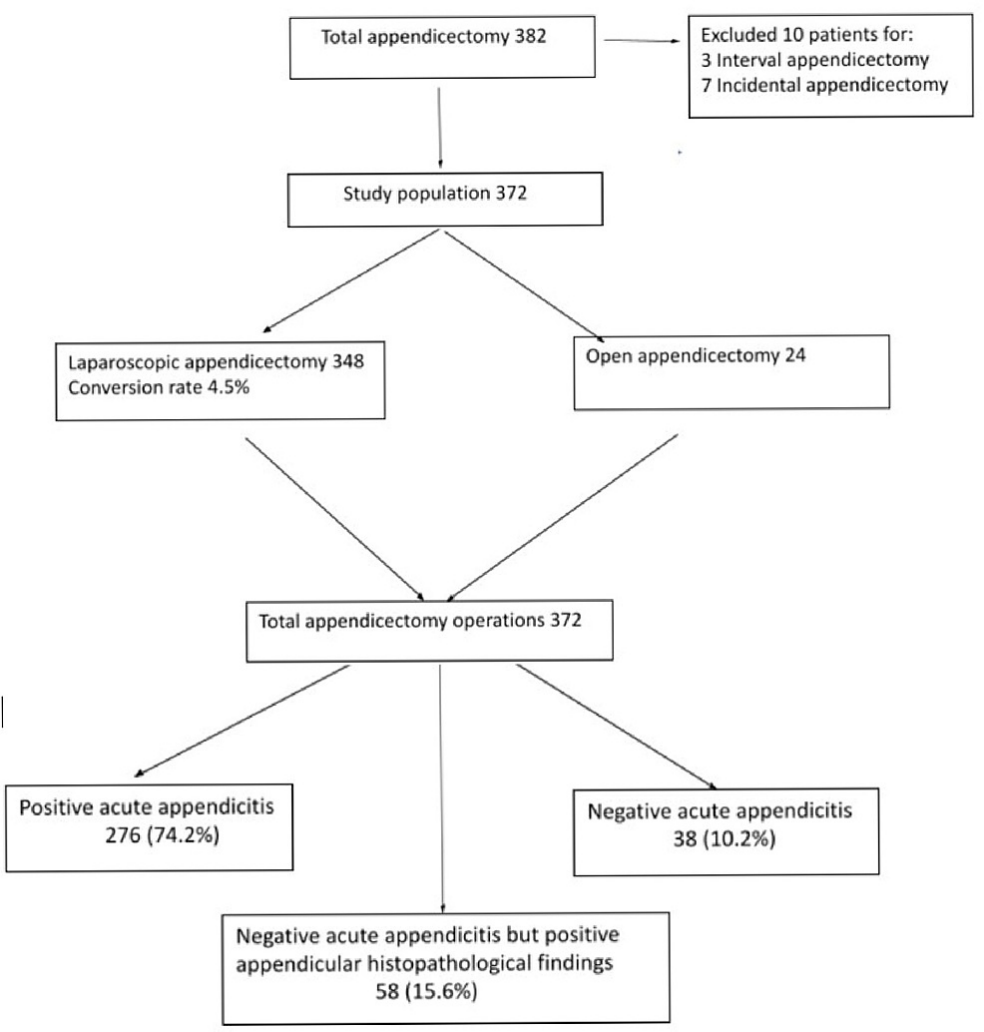
Summary flowchart of the results

A total of 382 appendicectomies were performed during the study period. We excluded 10 patients who underwent incidental (7) and interval (3) appendicectomies (Figure 1). The data of 372 patients who had emergency appendicectomy were analyzed. The median age was 27 (5-94) years, with 179 males and 193 females. The median ASA grade was 2 (1-4), and the mean Charlson comorbidity index was 0.6 (0-8). The median duration of symptoms was two days, and the median waiting time from admission to surgery was one day. The mean admission WBC, CRP, and serum bilirubin levels were 12,600 (3,000-38,000) per mm^3^, 66.9 (1-323) mg/L, and 12.7 (4-38) µmol/L, respectively.

Fifty percent of the patients in the study had imaging investigations done, with 22.6% undergoing USS and 27.4% having CT scans done. No patient had both imaging modalities done in this study. CT scan confirmed acute appendicitis in 95.1% of cases, while USS confirmed acute appendicitis in only 14.1% of the patients scanned. CT scan was concordant with positive acute appendicitis in 98% of the cases, while USS was only in agreement with 46% of the patients who had this imaging modality. It is worthy of note that four of the five patients whose CT scan showed no acute appendicitis were eventually confirmed to have acute appendicitis on histology. On the other hand, 17 of the 45 patients whose USS showed no evidence of inflammation had histological confirmation, and 11 of the 27 patients whose appendices were not visualized on USS all had confirmed acute appendicitis on histology.

The surgical approach was laparoscopic appendicectomy in 93.5% of the patients, with our conversion rate to open of 4.6%. Histopathological analysis showed acute appendicitis with infiltration of the appendicular wall with polymorphonuclear inflammatory cells in 74.2% of the patients, with 25.8% of the specimen showing no evidence of acute inflammatory process in the specimens. However, 89.8% of the appendicular specimen exhibited one form of microscopic pathological findings, while only 10.2% were devoid of any pathology in the specimens. The distribution of the pathological findings in the appendices is shown in Table 1.

**Table 1:** The distribution of the histopathological findings of the appendix

Appendicular Pathology	Number	Percentage (%)
Acute appendicitis	265	71.2
Appendicular tumour only	3	0.8
Appendicular faecolith only	30	8.1
Appendicular worms only	1	0.3
Appendicular lymphoid hyperplasia	6	1.6
Acute appendicitis and tumour	6	1.6
Acute appendicitis and worms	4	1.1
Appendicular fibrosis and lumen obliteration	15	4
Chronic appendicitis	3	0.8
Endometriotic infiltration of appendix	1	0.3
Normal histology	38	10.2

Therefore, our NAR-SDC was 10.2%, and NAR-STC was 25.8%. NAR was significantly higher in female than male patients (39.4% versus 11.1%; p-value 0.0001). About 16.3% of the patients with confirmed acute appendicitis had complicated pathology such as appendicular gangrene and/or perforation. The mean length of hospital stay was 1.9 (0-12) days. Patients with confirmed acute appendicitis stayed in the hospital slightly longer than those with negative appendicectomy (p-value 0.034).

Table 2 shows the demographic characteristics of patients who underwent appendectomy. There was a significant discordance in 23 patients between the surgeons' intraoperative findings of acute appendicitis and the final histopathological findings of appendicular wall infiltration with polymorphonuclear cells (299 versus 276; p-value 0.0001).

**Table 2:** Demographic and clinicopathologic characteristics of patients who had an appendectomy

Mean Age (yrs) ± SD		32.82 ± 19.52
Gender	Male (%)	179 (48.1%)
	Female (%)	193 (51.9%)
ASA Classification	1 (%)	127 (34.1%)
	2 (%)	218 (58.6%)
	3 (%)	24 (6.5%)
	4 (%)	8 (0.8%)
Median duration of symptoms (day) ± SD		2.00 ± 2.35
Median waiting time to surgery (day) ± SD		1.00 ± 0.72
Mean total white blood cells		12.6 ± 4.69
Mean C-reactive protein		66.9 ± 22.5
Mean total serum bilirubin		12.7 ± 5.32
Radiological investigations	USS (%)	84 (22.6%)
	CT Scan (%)	102 (27.4%)
	No radiological investigation (%)	186 (50.0%)
Histology of the appendix	Presence of acute inflammation (%)	276 (74.2%)
	Absence of acute inflammation (%)	96 (25.8%)
Pathology of the appendix	Acute appendicitis (%)	265 (71.2%)
	Appendicular tumour only (%)	3 (0.8%)
	Appendicular faecolith only (%)	30 (8.1%)
	Appendicular worms only (%)	1 (0.3%)
	Appendicular lymphoid hyperplasia (%)	6 (1.6%)
	Acute appendicitis and tumour (%)	6 (1.6%)
	Acute appendicitis and worms (%)	4 (1.1%)
	Appendicular fibrosis and lumen obliteration (%)	15 (4.0%)
	Chronic appendicitis (%)	3 (0.8%)
	Endometriotic infiltration of appendix (%)	1 (0.3%)
	Normal histology (%)	38 (10.2%)
Postoperative complications (%)		23 (6.2%)
Mean length of hospital stay± SD		1.89 ± 1.43
Postoperative readmission (%)		28 (7.5%)

Table 3 shows the details of 23 patients with disagreement between the intraoperative and the histopathological findings.

**Table 3:** Details of patients with discordance between the intraoperative and the histopathological findings M: Male, F: Female

Age	Sex	Grade of surgeon	Intraoperative findings	Histopathological reports
17	F	Registrar	Inflamed appendix	Endometriotic infiltration of appendix
16	M	SHO	Mildly inflamed appendix	Appendicular faecolith
8	F	Registrar	Acute appendicitis at distal part	Faecolith obstruction & congestion
25	M	Consultant	Acutely inflamed appendix	Faecolith obstruction & congestion
16	F	Registrar	Early acute appendicitis	Normal histologic findings
16	F	Registrar	Mildly congested appendix	Normal histologic findings
24	M	Registrar	Mildly inflamed appendix at tip	Normal histologic findings
21	F	Registrar	Acutely inflamed appendix	Normal histologic findings
36	M	Registrar	Congested inflamed appendix	Normal histologic findings
20	F	Registrar	Mildly inflamed appendix	Normal histologic findings
7	F	Registrar	Acute appendicitis	Lymphoid hyperplasia
14	F	Registrar	Inflamed tip of the appendix	Lymphoid hyperplasia
18	F	Registrar	Mildly inflamed appendix	Appendicular faecolith & congestion
26	F	Registrar	Mildly inflamed appendix	Appendicular faecolith & congestion
13	F	Consultant	Acutely inflamed appendix	Lymphoid hyperplasia
45	F	Consultant	Inflamed fibrotic appendix	Fibrotic appendix with lumen obliteration
28	F	Registrar	Inflamed distal appendix	Well differentiated neuroendocrine tumour
18	M	Consultant	Mildly inflamed appendix	Appendicular faecolith
13	F	Registrar	Inflamed congested appendix	Lymphoid hyperplasia
19	M	Registrar	Acute appendicitis & adhesions	Appendicular faecolith
43	M	Registrar	Inflamed appendix with faecolith	Embedded faecolith in appendix wall
16	F	Registrar	Acute appendicitis with omentum	Appendicular faecolith and mucositis
25	M	Registrar	Acute appendicitis	Appendicular congestion with faecolith

The cohort's postoperative complication and surgical readmission rates were 6.2% and 7.5%, respectively. The distribution of the postoperative complications and reasons for postoperative readmissions are listed in (Tables 4, 5), respectively. There was no significant difference in the postoperative complications between patients with confirmed acute appendicitis and the negative appendicectomy group (p-value = 0.132). However, the postoperative readmission was significantly higher in the negative appendicectomy group (p-value = 0.0001).

**Table 4:** Distribution of postoperative complications

Complication	Number
Wound infection	7
Postoperative intra-abdominal collection	3
Postoperative pain	3
Postoperative vomiting and diarrhoea	2
Postoperative constipation	2
Postoperative small bowel obstruction	1
Postoperative UTI	1
Postoperative fever	1
Stitch sinus	1
Postoperative urinary retention	1
Abdominal wall haematoma	2
Total (%)	23 (6.2%)

**Table 5:** Reasons for postoperative readmission NSTMI: Non-ST elevated Myocardial infarction

Reason	Number
Recurrent abdominal pain	13
Wound infection	6
Intra-abdominal collection	3
Constipation	2
Postoperative fever	1
Postoperative small bowel obstruction	1
Postoperative urinary retention	1
NSTEMI	1
Total (%)	28 (7.5%)

Table 6 shows the univariate analysis for factors predicting negative appendicectomy. Student t-test analysis comparing the mean of the continuous variables between the cohort with acute appendicitis and negative appendicectomy showed that patients with negative appendicectomy were younger (p-value 0.0001), had lower mean total WBC (p-value 0.014), lower CRP (p-value 0.0001), and lower total serum bilirubin (p-value 0.0001) levels on admission. Isolated serum bilirubin levels were significantly higher in the complicated compared to the non-complicated acute appendicitis groups (p-value 0.0001). Chi-square test univariate analysis identified variables such as younger age, female gender, lower ASA grade, lower Charlson CI, duration of symptoms more than three days, admission total WBC ≤ 11,000/mm^3^, CRP ≤ 5 mg/L, total serum bilirubin ≤ 20 µmol/L as significant predictors of negative appendicectomy. 

**Table 6:** Univariate analysis for factors predicting negative appendicectomy ASA: American Society of Anaesthesiologists, CI: comorbidity index, WBC: white blood cell, CRP: C-reactive protein

Variables		Total number	Acute appendicitis	Negative appendicectomy	P-value
Age group	≤15 years	76	61	15	0.0001
	16-45 years	205	125	79	
	>46 years	91	89	2	
Gender	Male	179	159	20	0.0001
	Female	193	117	76	
ASA group	ASA ≤2	345	250	95	0.006
	ASA >2	27	26	1	
Charlson CI group	CCI of 0	275	188	87	0.0001
	CCI > 0	97	88	9	
Symptom duration	Duration ≤3 days	288	254	34	0.0001
	Duration >3 days	84	22	62	
Waiting time to surgery	Waiting time ≤1 day	329	247	82	0.282
	Waiting time >2 days	43	29	14	
Admission WBC	WBC ≤11 X 10^3^ / mm^3^	144	83	61	0.0001
	WBC > 11 X 10^3^ / mm^3^	228	193	35	
Admission CRP	CRP ≤5 mg /L	75	33	42	0.0001
	CRP >5 mg /L	297	243	54	
Admission Bilirubin	Bilirubin ≤20 µmol/L	246	251	95	0.008
	Bilirubin >20 µmol/L	26	25	1	
Imaging investigation done	Yes	186	136	50	0.636
	No	186	140	46	
Grade of surgeons	Middle grade	295	215	80	0.037
	SHO	7	3	4	
	Consultant	70	58	12	
Time of surgery	Morning session	166	125	41	0.061
	Afternoon session	127	86	41	
	Night session	79	65	14	

All the statistically significant variables predicting negative appendicectomy were subjected to a multivariate logistic regression analysis calculating the OR and 95% CI. Female gender, duration of symptoms more than three days, lower total WBC were the independent predictors of negative appendicectomy (Table 7).

**Table 7:** Multivariate analysis of factors predicting negative appendicectomy ASA: American Society of Anaesthesiologists, CI: comorbidity index, WBC: white blood cell, CRP: C-reactive protein

Variables	Adjusted OR (95% CI)	P-value
Age group: ≤40 yrs. versus >40 yrs.	0.439 (0.129 - 1.489)	0.186
Gender: Female versus Male	5.400 (2.453 – 11.885)	0.0001
ASA group: ASA 1/2 versus 3/4	0.248 (0.012 - 4.968)	0.362
Charlson CI (CCI) group: CCI 0 versus CCI>0	2.592 (0.700–9.601)	0.154
Symptom duration: >3 days versus ≤3 days	18.710 (8.369–41.828)	0.0001
Admission WBC: ≤11,000/mm^3^ versus >11,000/ mm^3^	0.364 (0.177–1.752)	0.006
Admission CRP: ≤5 mg/L versus >5 mg/L	0.486 (0.215–1.096)	0.082
Admission Bilirubin: ≤ 20µmol/L versus >20µmol/L	0.155 (0.010–2.483)	0.188
Grade of surgeons: Registrar versus Consultants	0.171 (0.007–4.360)	0.285

## Discussion

The issue of negative appendicectomy is still a significant challenge in the surgical management of patients suspected of acute appendicitis. The reported negative appendicectomy rates vary widely in the published literature [2,5,7]. Unfortunately, there is no widely agreed-on standard definition of a negative appendicectomy as it has been used to mean a macroscopically and/or histologically normal appendix [5,7,8]. Mariadason et al. [7] submitted that the use of NAR is a flawed quality metric in the management of acute appendicitis as the reported rates are highly subjected to the definition of what a negative appendicectomy was considered to be in any particular setting. Therefore, using a stringent definition of acute appendicitis lowers the NAR; however, changing histological criteria for diagnosing inflamed appendix can raise the NAR by as much as 3%-6% in each case [7]. The predominance of the male population in some published studies also may have accounted for the lower NARs reported [7].

Our NAR using strict criteria (NAR-STC) was 25.8%, but when we used more liberal standard criteria, the rate (NAR-SDC) was much less at 10.2%. These figures are compared favourably with reported NARs in the literature [5,9,11-13]. Unlike many other studies reporting on NARs where there tends to be a preponderance of the male gender population, both males and females were equally represented in the current study. This equal distribution has eliminated the potential gender bias. Our finding of a significantly higher NAR in female than male patients (39.4% versus 11.1%) is not new as this has been widely supported by other authors [2,8,14]. Patients with NAR-STC, with no histological inflammatory process in their appendicular specimen but with other confirmed histological pathology may have developed their preoperative symptoms due to those pathological processes and will probably still require their appendix to be removed. Despite the absence of infiltration by the polymorphonuclear inflammatory cells, the findings of an appendicular tumour, faecoliths, and worms are significant. They should still be considered positive findings in the appendicular specimens. Therefore, these patients should be excluded from the negative appendicectomy group as they will most likely need to have their appendix removed anyway because of these significant pathological findings.

The presence of appendicular lymphoid hyperplasia, lumen fibrosis, and obliteration are non-specific and may indicate a recent or past inflammation of the appendix. The clinical significance of these pathological findings of the appendix is not well known. However, knowing that most acute appendicitis and appendicolic are triggered by a luminal obstruction, one can easily explain away the role that the presence of tumours, faecoliths, worms, lymphoid hyperplasia, and luminal fibrosis may play in causing the patients' symptoms of right iliac fossa pain. The presence of marked lymphoid hyperplasia has been responsible for appendicular obstruction leading to increased luminal pressure and contributing to symptoms in children [1,15-17]. Seventeen of the 23 patients with intraoperative findings of acute appendicitis but with negative histopathologically proven acute inflammation indeed had other pathological findings in the appendicular specimens that may have caused their presenting symptoms anyway. Only six of these patients had utterly normal histopathological findings in the appendix.

There is a recognized entity of viral appendicitis in the paediatric age group whose symptoms are known to be relieved by appendicectomy [2,15-17]. Acute appendicitis has been known to be preceded by non-specific viral-like illness before patients eventually present in the hospital. However, the microscopic examination of such an appendicular specimen will most likely yield an absence of acute inflammatory process and, therefore, be considered a negative appendicectomy. One of the features of viral appendicitis is the presence of lymphoid hyperplasia with or without viral epithelial changes such as appendicular mucosal ulceration and secondary bacterial infection [15,16,18]. Four out of 6 patients in this study with appendicular lymphoid hyperplasia on histology were in the paediatric age group, with 3 of them in their teens, the age at which one expected to have the highest appendicular lymphoid infiltration. Viral appendicitis is mostly self-limiting and can spontaneously resolve even without any form of treatment [15-19].

The use of imaging investigations and especially CT scans where indicated have been reported to lower the negative appendicectomy rates [2,7,10,20,21]. The use of CT scan in the diagnosis of appendicitis is widely utilized in the USA [2,20,21], and some authors have even advocated its routine use for diagnosis [2,19,21]. However, in Europe and particularly in the UK, imaging investigations are not routine but selective in the diagnosis of acute appendicitis [9,18]. Only 50% of the patients in the current study had either preoperative abdominopelvic USS or CT scan. Abdominopelvic USS and CT scans are selectively used where there is doubt about the diagnosis of acute appendicitis and/or where an alternative diagnosis is being considered. The diagnosis of acute appendicitis is still considered clinical on the ground of an adequate comprehensive history, clinical examination, and the consideration of relevant bedside and laboratory parameters. Repeated observation and clinical examination by an experienced surgeon is the key to making the diagnosis in those who are not so straightforward. It is well known that the utility of CT scan helps in the early and accurate diagnosis of acute appendicitis, reducing the delay in diagnosis with the morbidity associated with complicated appendicitis and valuable in excluding alternative diagnoses [7,21-23]. Tseng et al. [19] reported a NAR of 19.2% when CT scan was not used compared with 2.5% when CT scan was utilized in a large database of the American College of Surgeons National Surgical Quality Improvement Project (ACS-NSQIP). They concluded that imaging investigations, especially CT-scan, led to a significantly lower NAR. Coursey et al. [24], in another study looking at the role of CT scan in the management of acute appendicitis, reported that CT decreased the overall NAR from 16% to about 4%, a well desirable outcome. The reduction in the NAR was more noticeable and more observed in women within the 18-45 years age bracket, with a decrease from 42.9% to 7.1%. They observed that CT scans failed to significantly impact the NAR in men and women over the age of 45 years. However, in another study, even when CT scan was routinely used in their patients to diagnose acute appendicitis, Kim et al. [23] still reported a negative appendectomy rate of 7%. Preoperative imaging investigations were selectively utilized in this study as this is our usual practice. We generally engage the selective use of abdominopelvic USS in our institution for children and women of reproductive age with tentative diagnosis to avoid exposure to CT scan radiation and exclude gynaecological problems, respectively.

There have been reports of increased morbidity, prolonged hospital stay, and, on some occasions, increased mortality in patients who have had negative appendicectomy [2,8,13]. The findings in the present study do not support those claims as we did not find a significant difference in the morbidity rates between the two groups. Our patients with acute appendicitis stayed in the hospital slightly longer than the negative appendicectomy group. We believe that this prolonged hospital stay was due to postoperative complications in some patients with complicated appendicitis and for social reasons in some of the elderly patients who were waiting for placement for recovery after discharge. What we have noted in this study was the significantly higher postoperative readmission episodes among the negative appendicectomy group compared with those with acute appendicitis. The high proportion of the patients with negative appendicectomy readmitted was because of ongoing non-resolving abdominal pain even after their appendix had been removed. This finding is particularly relevant and may indicate that the original diagnosis was missed and/or point to the existence of an alternative cause for their pain. We reported no mortality in this study.

There are contentions with varying arguments about handling a normal-looking appendix at diagnostic laparoscopy for right lower abdominal pain [5,9,10]. There are wide variations in practice with divided opinions on both sides of the Atlantic concerning managing a normal appendix at laparoscopy [5,6,9,24]. Jaunoo et al. [25] reported that about 73% of the surgeons surveyed in America and Europe would remove a macroscopically normal appendix at diagnostic laparoscopy for suspected appendicitis provided no other abnormal finding. The authors advocated that appendectomy be performed in the absence of an alternative explanation for the patient's symptoms because almost a third of macroscopically normal appendices are found to be inflamed on histological analysis [25]. We in our institution generally adopt, like many other authors, the acceptable practice that a macroscopically normal appendix found at laparoscopy be left alone, especially if there is another reason(s) to account for the patient symptoms [5,9,10,26]. However, we do advocate removing a normal appendix at laparoscopy as such may be harbouring microscopic appendicitis and/or other pathology that may be responsible for the patient's symptoms, a view supported by many other authors [5,13,26,27]. Turner and Lightwood [27] reported that 61% of British and Irish surgeons would remove a macroscopically normal appendix at laparoscopy. The justification for this approach has been demonstrated in the current study where 15.6% of our patients had identifiable pathology in the appendicular specimens, some of them very significant without the histopathological features of acute appendicitis. Nine patients in this study were found to be harbouring tumours in their appendix specimen, and three of those were without histological features of acute inflammation.

Our study has identified certain independent predictors of negative appendicectomy, including female gender, prolonged duration of symptoms, and normal total white blood cells on admission. These findings are supported by other published reports demonstrating that young patients, female gender, having a lower proportion of polymorphonuclear cells or normal WBC and lower heart rate could be considered as independent predictors of negative appendicectomy [2,9,14,19]. Univariate analysis showed a significantly higher NAR in women than men, and in fact, the female gender is an independent predictor of negative appendicectomy. This study showed that being female increased the risk of a negative appendicectomy by almost four folds when compared with the male patients. Generally, the NAR in women of reproductive age is reportedly higher because of various gynaecological problems that may mimic similar symptoms to acute appendicitis [2,5,6,9,13,14]. A normal WBC on admission in this study was highly predictive of a negative appendicectomy. Similar findings have been variously reported in the published literature [2,12,14]. However, one must exercise caution as a very small group of young, normally fit patients, especially men, have been anecdotally found to present with normal levels of WBC and/or CRP and yet were confirmed to have acute appendicitis at surgery and/or by histopathology. The admission CRP levels showed some promise in predicting negative appendicectomy on univariate analysis, but it was found not to be statistically significant on its own on multivariate logistic regression. Jeon [2] has also previously reported that CPR levels failed to relate to negative appendicectomy in his study. In a meta-analysis by Andersson [26], he reported that all clinical and laboratory parameters, when viewed individually, are poor discriminators for acute appendicitis, but when combined, they become a powerful tool in improving diagnostic certainty and reducing negative appendectomy rate. There have been conflicting reports of the discriminatory role of isolated hyperbilirubinemia in complicated acute appendicitis [12,23,26]. We found an isolated elevated serum bilirubin level to be somehow discriminatory of complicated acute appendicitis. Caution must be exercised at generalizing this finding as the number of our patients with isolated bilirubinaemia was small in this cohort.

There are limitations to this study, including the fact that it was a retrospective cohort study from a single institution with its potential bias. The inclusion of patients with different age groups and a wide range of surgeons with different levels of experience operating may have affected the decisions during the operation. We have noted some discordance between the surgeons' record of finding acute appendicitis during the operation and the final histological findings of an absent inflammatory process in the specimens. However, most of the patients had confirmed histological abnormalities in the appendicular specimens.

## Conclusions

Negative appendicectomy is still a significant problem in managing acute right lower abdominal pain patients. There is generally a lack of consensus and evidence-based guidelines on managing a normal appendix found at laparoscopy for right iliac fossa pain. We have identified independent predictors of negative appendicectomy as female gender, prolonged duration of symptoms, and normal total white blood cells on admission. , there may be a need to increase selective use of abdominopelvic USS/CT scan, according to gender and age group, to reduce the negative appendectomy rate. More importantly, a high index of suspicion for other confounding diagnoses and careful clinical skills by experienced clinicians may help to avoid negative appendicectomy.
